# Catheter body-surface fixation after transurethral prostate resection: A low-value nursing practice as evidenced in a randomized controlled trial

**DOI:** 10.1371/journal.pone.0350800

**Published:** 2026-06-04

**Authors:** Yanan Zhu, Qian Wang, Huiying Jia, Gaiyun Zhao, Yunpeng Lü, Xinhong Zhang, Haijing Dong

**Affiliations:** 1 Emergency Department, Qingdao Municipal Hospital, Qingdao, Shan Dong, China; 2 Department of Urology, Qingdao Municipal Hospital, Qingdao, Shan Dong, China; 3 Department of Cardiology, Qingdao Municipal Hospital, Qingdao, Shan Dong, China; 4 Department of Operating Rooms, Qingdao Municipal Hospital, Qingdao, Shan Dong, China; 5 Department of Nursing, Qingdao Municipal Hospital, Qingdao, Shan Dong, China; University of Washington, UNITED STATES OF AMERICA

## Abstract

This randomized controlled trial is aimed at evaluating whether external fixation of the urinary catheter to the body surface represents a low-value nursing intervention for patients undergoing transurethral resection of the prostate (TURP). A total of 208 patients who received indwelling urinary catheters after TURP in a tertiary hospital in Qingdao, China between June 2024 and May 2025 were randomly assigned to one of two groups: a nonexternal fixation group (n = 103) and an external body surface fixation group (n = 105). A between-group comparison of outcomes included postoperative hematuria, incidence of catheter-associated urinary tract infection (CAUTI), unplanned catheter removal, occurrence of urinary catheter-related meatal pressure injury (UCR-MPI), and associated economic costs. No significant differences were observed between the two groups in terms of postoperative hematuria or CAUTI incidence (P > 0.05). Unplanned catheter removal did not occur in either group. However, UCR-MPI occurred significantly more frequently in the external fixation group (9 patients) than it did in the nonexternal fixation group (1 patient) (P < 0.05). Additionally, the external fixation group incurred higher costs for personnel and consumables. External fixation of the urinary catheter to the body surface after TURP is associated with increased economic costs, reduced patient comfort, and a higher incidence of UCR-MPI, which indicates that it constitutes a low-value nursing practice. Nonexternal fixation appears to be a safe and effective alternative for post-TURP patients undergoing early mobilization.

## Introduction

Given the global backdrop of efforts by healthcare systems to enhance care value and efficiency, the identification and reduction of “low-value care” has become a critical imperative. Low-value care is typically defined as clinical practices that offer minimal benefit to patients, of which the potential risks or costs may outweigh the gains [1]. It is estimated that approximately 30% of healthcare services may be categorized as wasteful or of low value [2,3], consuming finite medical resources and potentially imposing unnecessary burdens on patients’ physical and psychological well-being [2–5]. Therefore, the prudent evaluation and optimization of routine practices within nursing are essential for advancing evidence-based practice and achieving optimal resource allocation.

Transurethral resection of the prostate (TURP) is the standard surgical procedure for treating benign prostatic hyperplasia [6,7]. Postoperative indwelling urinary catheterization is typically needed. However, urinary catheter-related meatal pressure injury (UCR-MPI) is a recognized complication, in which male patients are at increased risk because of their anatomical characteristics [8,9]. With the widespread adoption of enhanced recovery after surgery (ERAS), early postoperative ambulation for TURP patients has become an aspect of standard nursing care [10]. This presents a dilemma in catheter management regarding the proper securing of the catheter to facilitate patient mobility while avoiding injuries caused by improper fixation.

Currently, external fixation of the catheter to the patient’s body surface, such as the thigh or abdominal wall, is common in clinical practice; however, no unified standard exists for this procedure [11]. Existing evidence has suggested that improper fixation can lead to catheter dislocation, friction, and pressure on the urethral mucosa, thereby increasing patient discomfort, injury, and infection risk [12,13]. Furthermore, issues such as the curling and detachment of adhesive tape add to the nursing workload and material costs [14]. Notably, despite these potential problems, “external catheter fixation to the body surface” has not been explicitly listed as a low-value nursing practice on either the list developed in China on the basis of clinical nursing practice guidelines [15] or on lists released through “choosing wisely” campaigns in countries such as the United States, the Netherlands, and Canada [16–18]. This gap suggests a lack of rigorous evidence from high-level randomized controlled trials for evaluating this routine practice [19].

Consequently, this study aimed to systematically assess the practical value of the external body surface fixation of urinary catheters—a widely used nursing measure—in post-TURP patients through a randomized controlled trial. Guided by the core defining attributes of low-value care (i.e., care backed by insufficient evidence of effectiveness, where harm outweighs benefit, care that is not cost-effective, and that which does not align with patient values and preferences) [20], two strategies, namely, “external body surface fixation” and “nonexternal body surface fixation”, are compared in this research. The comparison is focused on postoperative bleeding, the incidence of catheter-associated urinary tract infection (CAUTI), unplanned extubation rates, UCR-MPI incidence, and economic costs. The goal is to provide clear evidence-based guidance for clinical practice and to optimize the existing postoperative urinary catheter management protocols.

## Methods

### Study design and participants

All patients in this study gave informed consent, and ethical approval was obtained from Qingdao Municipal Hospital (2024-LW-047). Written consent was obtained from all patients before being enrolled in the studies,and abide by the principles of the Helsinki Declaration.Clinical trial number: ChiCTR2500111902.

**Fig 1:**
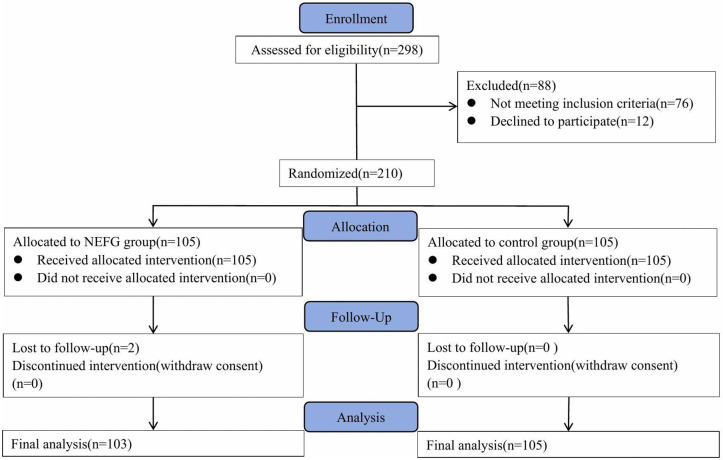
CONSORT diagram of the trial.

A total of 210 patients who had undergone TURP and who required postoperative indwelling urinary catheterization between June 2024 and May 2025 were recruited. Participants were randomly assigned to one of two groups: the catheter external body–surface fixation group (n = 105) or the catheter nonexternal fixation group (n = 105). During the study period, two patients in the nonexternal fixation group required early catheter removal for clinical reasons that were unrelated to the intervention, and they were thus excluded from the final analysis. Consequently, 208 patients completed the study (external fixation group: n = 105; nonexternal fixation group: n = 103). Patient recruitment, randomization, and follow-up details are summarized in Fig 1.

### Sample size calculation

The sample size was calculated during the study design phase prior to patient enrollment. The sample size was calculated on the basis of the primary outcome (UCR-MPI, grade ≥I by postoperative day 3). The baseline incidence (10.7%, 13/121) was derived from our center’s retrospective TURP patient data. We hypothesized that nonexternal fixation would reduce this to 1.0%. The use of two independent proportions formulas with α = 0.05 and power = 80% required a minimum of 88 patients in each group. Accounting for a 10% dropout, we enrolled 105 patients per group (a total of 210), with the final 208 patients providing sufficient power.

**Inclusion criteria:**

(1) The patients should have been diagnosed with benign prostatic hyperplasia (BPH) that met the surgical indications outlined in the national “Guidelines for the Diagnosis and Treatment of Benign Prostatic Hyperplasia”; (2) they should have a negative preoperative urine culture; (3) all surgeries should have been performed by the same medical team to standardize the surgical technique; (4) they should not have limb mobility or communication disorders; (5) their prostatic tissue hyperplasia should be pathologically confirmed postoperatively; and (6) they should provide a written informed consent.

**Exclusion criteria:**

The patients should not have (1) preoperative coagulation disorders, infection at the puncture site, acute or chronic pain conditions, or drug addiction; (2) concurrent presence of other urological diseases; (3) history of prolonged indwelling urinary catheterization; (4) known allergy to medical adhesives; and (5) planned indwelling catheter time of less than 1 day or more than 3 days post-operatively.

### Randomization and intervention

Eligible patients were randomly assigned to either the EFSG or the NEFG at a 1:1 ratio. A computer-generated random sequence was prepared by an independent researcher who was not involved in patient recruitment. Sequentially numbered, sealed opaque envelopes were used to ensure allocation concealment. Upon admission, the patients were assigned to groups by opening the corresponding envelope. Both groups received standardized perioperative care in adherence with enhanced recovery after surgery (ERAS) principles.

#### 1. Catheter External Body-Surface Fixation Group (EFSG).

For the patients in this group, a urinary catheter was secured to the patient’s inner thigh using the “high-lift platform” method with 3 M™ medical elastic adhesive tape, as described in previous evidence-based practice recommendations [21]. This technique is aimed at enabling full leg extension that does not result in tension on the catheter. When ambulating, the urine collection bag was attached to the patient’s waistband using an “S”-shaped hook or a cord to ensure that the bag remained below the bladder level and that the catheter maintained a natural, slack “N”-shaped configuration to minimize tension and drag.

#### 2. Catheter Nonexternal Fixation Group (NEFG).

For the patients in this group, the catheter was not fixed to the skin using adhesive tape. Rather, as part of the intervention protocol, patients wore a modified hospital gown featuring discrete openings on both trouser legs (approximately 5–10 cm above the knee). The urine collection tubing was routed through this opening, and the bag itself was secured to the trouser leg. This design allowed the catheter to hang naturally without external fixation, which minimized tension on the external genitalia and facilitated easy observation of urine output during ambulation. When in bed, standard care was maintained with the collection bag positioned on the proximal side rail.

To ensure patient safety and protocol adherence during early mobilization, nurses in both groups provided patients with standardized education. This included instructing the patients to empty the collection bag before getting out of bed, to ambulate slowly, avoiding vigorous movements (e.g., long strides, brisk walking), and to always keep the collection bag and tubing below the bladder level to prevent backflow and infection.

### Research team

A dedicated research team of 11 members was established to ensure rigorous implementation and data integrity. The team comprised three urologists (responsible for patient screening and ongoing clinical oversight), five registered nurses (responsible for patient assessment, intervention delivery, and data recording), two postgraduate research students (responsible for data collection, entry, and preliminary analysis), and one nurse manager (responsible for overall study coordination, process supervision, and evaluation of intervention fidelity).

### Outcome measures

The following primary and secondary outcome measures were assessed to compare the efficacy and economic impact of the two catheter management strategies, with a focus on verifying the core characteristics of low-value care (insufficient evidence, harm exceeding benefit, lack of cost-effectiveness) [22].

### Primary outcome

Incidence of UCR-MPI (Grade≥I) by postoperative day 3. This outcome was selected as the primary measure because UCR-MPI is the most direct complication of catheter fixation and a direct reflection of the “harm exceeding benefit” feature of low-value care [8,22].

### Secondary outcomes

#### (1) Severity of postoperative hematuria.

**Fig 2:**
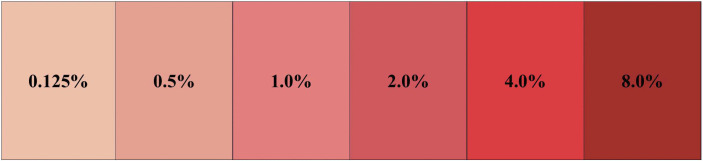
Six-color scale.

Hematuria was objectively assessed using the validated Continuous Bladder Irrigation (CBI) Drainage Fluid Color Assessment Tool [23]. This tool features a six-color scale corresponding to the following specific blood concentrations: 8%, 4%, 2%, 1%, 0.5%, and 0.125%. See Fig 2 for details.

Urine samples were collected by trained research nurses (blinded to group allocation) at three standardized time points: (1) during bed activity on the day of surgery; (2) during the first postoperative ambulation; and (3) during the second postoperative ambulation. The highest color grade that corresponded to the drainage fluid was recorded for analysis. Prior to study initiation, to ensure assessment consistency, three research nurses completed a training program that including both tool operation and simulated case practice, with a required interrater kappa coefficient ≥0.85.

This outcome was selected because hematuria severity reflects urethral mucosal irritation or injury caused by catheter movement, which is closely related to fixation stability, which is one of the key differences between the two intervention strategies [21].

#### (2) Incidence of catheter-associated urinary tract infection (CAUTI).

CAUTI was defined according to the Centers for Disease Control and Prevention’s (CDC) National Healthcare Safety Network (NHSN) criteria: (1) the presence of at least one urinary tract symptom (e.g., dysuria, frequency, suprapubic tenderness); (2) a positive urine culture (≧10⁵ colony-forming units [CFU]/mL of a single pathogenic organism); and (3) no other identified source of infection.

Mid-stream urine samples were aseptically collected from all patients immediately prior to catheter removal on postoperative day 3. The specimens were sent to the clinical microbiology laboratory for routine culture and antimicrobial susceptibility testing. The incidence of CAUTI was calculated as the number of patients with CAUTI divided by the total number of participants in each group.

This outcome was included because improper catheter fixation can lead to urethral mucosal damage or retrograde infection, which is a key indicator of “potential harm” in low-value care [12,20].

#### (3) Unplanned extubation rate.

Unplanned extubation was defined as any unintended removal of the urinary catheter, including accidental dislodgement (e.g., due to inadequate fixation) or patient self-removal. The rate was calculated using the international standard formula for healthcare-associated adverse events [19]:

Unplanned extubation rate = (total number of unplanned extubation events/total number of catheter-days in the group) × 1000, where “total number of catheter-days” refers to the sum of the indwelling catheter days for all patients in the group. This outcome was selected because unplanned extubation is associated with increased recatheterization risk, prolonged hospital stay, and higher healthcare costs, which are key markers of the “lack of cost-effectiveness” that characterizes low-value care [20].

#### (4) Urinary catheter-related meatal pressure injury (UCR-MPI).

UCR-MPI was assessed by blinded research nurses on a daily basis until catheter removal. The diagnosis and grading (I-IV) were based on validated criteria specifically for male postoperative urological patients [8]:

Grade I: Nonblanchable erythema with intact urethral meatal skin/mucosa;

Grade II: Partial-thickness skin/mucosal loss at the urethral meatus;

Grade III: Full-thickness skin and urethral mucosal injury involving <2 cm of the urethra;

Grade IV: Full-thickness injury involving ≥2 cm of the urethra or deep tissue involvement.

Both the incidence (proportion of patients with Grade≥I UCR-MPI) and severity (highest grade recorded) were documented.

As the primary outcome, UCR-MPI directly reflects the physical harm that is caused by catheter fixation, which is central to evaluating whether the intervention meets the “harm exceeding benefit” definition of low-value care [20].

### Total economic costs (personnel + consumables)

Economic costs were evaluated from the hospital perspective using a microcosting approach, which is focused on the detailed tracking of direct resource consumption for healthcare interventions. Consistent with the study’s objective of identifying low-value care (i.e., lack of cost-effectiveness) [24], costs were categorized into two components: personnel (energy) cost and consumables cost.

Personnel cost was operationalized on the basis of the frequency of catheter securement-related nursing interventions, including reapplication for dislodgement, position adjustment, and the management of adhesive curling or detachment. All interventions were prospectively recorded with documentation of key details (e.g., problem addressed) to ensure data accuracy and traceability.

Since intervention duration data were not collected, the frequency of securement-related interventions was used as the core indicator to quantify the relative nursing workload burden. This choice is supported by evidence: Chambers et al. [25] confirmed a positive correlation between nursing intervention frequency and labor input, and Lima et al. [26] further reported that intervention frequency serves as a reliable surrogate for human resource consumption, thereby providing a methodological basis for cost assessment when duration data are unavailable and when data are aligned with clinical practice and evidence-based principles.

The EFSG: Consumable costs were calculated on the basis of the use of 3 M™ medical elastic adhesive tape (standardized to 5 cm × 7 cm strips) for catheter fixation. The unit price was determined by the hospital’s official procurement records (￥1.56 per strip), and total cost was computed as the product of strips used per patient and unit price.

NEFG: No additional consumables (e.g., adhesive tape) were used for catheter fixation, which resulted in a cost of￥0.

Total economic cost was defined as the sum of the personnel (reflected by securement-related intervention frequency) and consumables costs. This composite indicator is used to comprehensively assess the resource utilization efficiency of the two catheter management strategies, directly supporting the study’s goal of identifying low-value care [20].

### Handling missing data

For outcome measures with missing data (e.g., missed hematuria assessment due to patient temporary unavailability), multiple imputation (MI) with 10 imputed datasets was used. The variables included in the imputation model were group allocation, age, preoperative prostate volume, and other relevant baseline characteristics.

### Assessment quality control

All outcome assessors (research nurses) were blinded to group allocation. Blinding was maintained by having a separate research team member (who was not involved in outcome assessment) manage group allocation and intervention implementation. Regular quality checks (10% of the assessments were randomly reviewed by the nurse manager) were conducted throughout the study to ensure adherence to the assessment protocols and maintain blinding integrity.

### Statistical analysis

Epidata 3.0 software was used for double-entry data, and SPSS 21.0 software was used for statistical analysis. The normally distributed data are represented as the mean± standard deviation, the nonnormally distributed data are expressed as the median and interquartile range, and the count data are described as the frequency and constitutive ratio; intergroup comparisons were performed via t tests, chi-square tests, and rank-and-rank tests, with a significance level of α = 0.05.

## Results

### Baseline characteristics

There were no statistically significant differences in demographic, clinical, or surgical characteristics between the two groups at baseline (all P > 0.05), indicating adequate randomization and comparability (Table 1).

**Table 1:** Baseline characteristics of patients in the EFSG and NEFG groups (n = 208).

Sports event		EFSG (N = 105)	NEFG (N = 103)
Age (years)		70.71 ± 8.16	71.99 ± 7.10
BMI		24.8 (22.49,27.15)	24.69 (22.92,26.88)
Religious belief	Yes	103 (98.1%)	103 (100%)
	No	2 (1.9%)	0 (0%)
Educational attainment	Below primary level	18 (17.1%)	11 (10.7%)
	junior high school	44 (41.9%)	39 (37.9%)
	High school/vocational high school/secondary school	30 (28.6%)	28 (27.2)
	College and above	13 (12.4%)	25 (24.3)
Smoking history	Never smoked	79 (75.2%)	77 (74.8%)
	Currently smoking	22 (21.0%)	21 (20.4%)
	has given up smoking	4 (4.5%)	5 (4.9%)
Preoperative prostate volume		70.00 (41.24,91.50)	70.00 (46.19,87.05)
Preoperative PSA (ng/ml)		4.32 (2.45,6.68)	4.88 (2.10,7.00)
Preoperative residual urine volume (RUV) (ml)		50.00 (35.00,90.00)	59.00 (43.00,89.50)
Number of types of chronic diseases combined (types)		3 (2,6)	4 (2,8)
Anesthesia	general anesthesia	22 (21%)	15 (14.6%)
	epidural anesthesia	49 (46.7%)	45 (43.7%)
	Combined spinal-epidural anesthesia	34 (32.4%)	43 (41.7%)
Length of surgery (min)		89.00 (58.50,112.50)	80.00 (62.00,100.00)
Postoperative bladder irrigation time (h)		19.00 (16.00, 20.25)	18.00 (16.00,21.00)
Postoperative analgesic and antispasmodic drugs	Yes	65 (61.9%)	55 (53.4%)
	No	40 (38.1%)	48 (46.6%)
Preoperative International Prostate Symptom Score		23 (20,27)	23 (21,26)

Notes: BMI, body mass index; PSA, prostate-specific antigen*.*

### Primary outcome

#### Incidence of Urinary Catheter-Related Meatal Pressure Injury (UCR-MPI).

As the primary outcome, the incidence of grade ≥I UCR-MPI was significantly greater in the EFSG than it was in the NEFG (P = 0.010; Table 2). Specifically, 9 patients (8.57%) in the EFSG developed Grade I UCR-MPI, whereas only 1 patient (0.97%) in the NEFG had Grade I UCR-MPI. No grade II–IV UCR-MPI was observed in either group.

**Table 2:** Comparison of outcomes between the EFSG and NEFG groups (N = 208).

Variables	EFSG (n = 105)	NEFG (n = 103)	Test Statistic	P value
UCR-MPI			6.563^a^	*P* = 0.010
Class I UCR-MPI, n(%)	9 (8.57)	1 (0.97)		
No UCR-MPI, n(%)	96 (91.43)	102 (99.03)		
CAUTI			0.267^a^	*P* = 0.783
No, n(%)	97 (92.38)	97 (94.17)		
Yes, n(%)	8 (7.62)	6 (5.83)		
Unplanned extubation rate, n(%)	0(0.0)	0(0.0)	–	*–*
Frequency of external catheter fixation problems, Median (IQR)	3 (2, 4)	0 (0, 1)	−9.191^b^	*P* = 0.001
Cost of medical elastic tape consumables (¥)	444.6	0	–	–
Blood Concentration (n, %) – Day of surgery			−0.297^b^	*P* = 0.767
0.125%	2(1.9)	4(3.9)		
0.5%	18(17.1)	13(12.6)		
1.0%	42(40.0)	46(44.7)		
2.0%	25(23.8)	26(25.2)		
4.0%	10(9.5)	11(10.7)		
8.0%	8(7.6)	3(2.9)		
Blood Concentration (n, %) – First postoperative day			−0.828^b^	*P* = 0.408
0.125%	0(0.0)	1(1.0)		
0.5%	58(55.2)	61(59.2)		
1.0%	33(31.4)	29(28.2)		
2.0%	9(8.6)	10(9.7)		
4.0%	2(1.9)	1(1.0)		
8.0%	3(2.9)	1(1.0)		
Blood Concentration (n, %) – Second postoperative day			−1.698^b^	*P* = 0.090
0.125%	0(0.0)	1(1.0)		
0.5%	71(67.6)	80(77.7)		
1.0%	26(24.8)	14(13.6)		
2.0%	8(7.6)	6(5.8)		
4.0%	0(0.0)	2(1.9)		
8.0%	0(0.0)	0(0.0)		

Notes: EFSG, external fixation group; NEFG, nonexternal fixation group; UCR-MPI, urinary catheter-related mental pressure injury; CAUTI, catheter-associated urinary tract infection; and IQR, interquartile range. Hematuria severity was assessed using the validated CBI Drainage Fluid Color Assessment Tool [23], with higher color scales indicating more severe hematuria. a) Chi-square test and b) Rank-sum test.

### Secondary outcomes

#### Catheter-Associated Urinary Tract Infection (CAUTI) incidence.

CAUTI occurred in 8 patients (7.62%) in the EFSG and in 6 patients (5.83%) in the NEFG. There was no statistically significant difference in the CAUTI incidence between the two groups (x² = 0.267, *P* = 0.783; Table 2). All positive urine cultures revealed single-pathogen colonization, with no severe infectious complications reported.

#### Postoperative hematuria severity.

Hematuria severity was assessed using the Continuous Bladder Irrigation (CBI) Drainage Fluid Color Assessment Tool at three time points: during bed activity on the day of surgery and during ambulation on the first and second postoperative days. The tool features six color scales corresponding to blood concentrations of 8%, 4%, 2%, 1%, 0.5%, and 0.125%. The distribution of hematuria grades was not significantly different between the two groups at any time point (all *P* > 0.05; Table 2). On the second postoperative day, 67.6% of the patients in the EFSG and 77.7% of those in the NEFG had mild hematuria (blood concentration≤1.0%), which is consistent with the expected recovery course after TURP.

#### Unplanned extubation rate.

No unplanned extubation events (accidental dislodgement or patient self-removal) occurred in either group during the study period. All the catheters were removed as planned on postoperative day 3, with no complications related to catheter retention or removal.

### Economic costs

#### Frequency of fixation-related nursing interventions.

The total number of fixation-related nursing interventions (e.g., retaping detached catheters, adjusting the fixation position, and managing adhesive issues) was 285 in the EFSG and 72 in the NEFG. The median number of interventions per patient was significantly greater in the EFSG (3 (2, 4) times) than it was in the NEFG (0 (0, 1) times) (*Z* = −9.191; *P* < 0.001; Table 2).

#### Consumables cost.

The EFSG incurred additional consumable costs for 3 M medical elastic adhesive tape. With a unit cost of ¥1.56 per strip (cut to 5 cm × 7 cm for fixation), the total cost of consumables for the EFSG was ¥444.6, while the NEFG had no such expenditure (Table 2).

## Discussion

In this randomized controlled trial, two urinary catheter management strategies—external body-surface fixation versus nonexternal fixation—are rigorously evaluated in patients who mobilize early following transurethral resection of the prostate (TURP). Framed within the conceptual model of low-value care, which is defined by insufficient evidence of effectiveness, harm that outweighs benefit, a lack of cost-effectiveness, and misalignment with patient preferences [20], this study addresses a notable evidence gap in postoperative nursing. Our findings indicate that routine external fixation of the urinary catheter, a common clinical practice, fulfills all four defining criteria of low-value care for this specific patient population.

### Alignment of the findings with low-value care attributes

#### Insufficient evidence of effectiveness.

The nonexternal fixation strategy did not lead to a higher incidence of postoperative hematuria, catheter-associated urinary tract infection (CAUTI), or unplanned catheter removal. These findings challenge conventional nursing guidance, in which secure external fixation is emphasized as essential for the prevention of catheter-related complications [13,24]. The existing guidelines, such as those from the British Association of Urological Surgeons and Nurses, are predominantly focused on patients who require long-term indwelling catheters and those with significant mobility restrictions [13]. In contrast, our study enrolled alerted, compliant patients who had undergone short-term catheterization (≦3 days) and who were mobilized early under an enhanced recovery after surgery (ERAS) protocol [10]. Within this context, the avoidance of adhesive fixation did not compromise catheter security or patient safety. As Howard et al. [19] contended, the lack of proven effectiveness for a routine practice is a primary indicator of its low value, particularly when a simpler alternative (e.g., natural suspension facilitated by a modified gown) demonstrates noninferior outcomes.

#### Harm that outweighs the benefits.

The superior outcomes observed in the nonexternal fixation group were further substantiated by a striking difference observed in the incidence of UCR-MPI: 8.57% in the external fixation group (EFSG) versus only 0.97% in the nonexternal fixation group (NEFG) (P = 0.010). This represents an approximately 9-fold reduction in risk for members of the NEFG, a discrepancy that is both statistically significant and clinically meaningful. Beyond the immediate reduction in iatrogenic mucosal injury, this finding addresses a critical gap in current perioperative care: routine external fixation, while common, fails to provide incremental benefits while introducing avoidable harm. Among early mobilized patients after TURP, for those who exhibit higher levels of physiological resilience and shorter catheter dwell times, external fixation induces unnecessary mechanical stress on the already compromised urethral mucosa, which indicates that it is a low-value form of care according to contemporary clinical standards.

This result is mechanistically sound: external fixation induces catheter curvature, which results in sustained lateral pressure on the urethral meatus and leads to mucosal ischemia and subsequent injury [8,27–30]. The TURP procedure itself compromises the urethral mucosal barrier [8], and the addition of a fixed catheter exacerbates mechanical trauma, particularly when faced with increased postoperative secretions and friction during ambulation. Conversely, the nonexternal fixation approach enables the catheter to assume a natural, tension-free position, which significantly reduces mechanical stress on the urethral mucosa. This design also enables patients to make minor self-adjustments for comfort, which aligns with the ERAS principle of promoting patient autonomy [10,21]. The clear evidence of greater physical harm without countervailing benefits reinforces the classification of routine external fixation as low-value care [20]. In summary, the 8.57% versus 0.97% difference in UCR-MPI incidence between groups provides compelling evidence for the discontinuation of routine external fixation for early mobilized patients following TURP. Future prospective studies that leverage larger sample sizes and longer follow-up periods are warranted to validate these findings across patient populations and healthcare settings.

#### Lack of cost-effectiveness.

External fixation incurred unnecessary healthcare costs without improving clinical outcomes. The direct consumable costs for adhesive tape totaled ¥444.6 for the external fixation group, an expense that is absent in the nonfixation group. More substantially, the nursing workload related to fixation management was markedly greater in the external fixation group than it was in the nonfixation group, with a median of 3 interventions per patient (e.g., for tape detachment or repositioning) compared with 0 in the nonfixation group. In an ERAS setting in which early and frequent mobility is encouraged [10], the need for repeated catheter resecurement increases nursing time and resource utilization [27]. Sacristán [31] and others have reported that cost-effectiveness is central to healthcare value assessment; in this study, the additional resources consumed by external fixation yielded no measurable patient benefit, which fulfills the criterion of a “lack of cost-effectiveness” [20].

### Misalignment with patient preferences and comfort

Qualitative feedback from the participants further highlights the low-value nature of routine external fixation. Patients in the external fixation group frequently reported discomfort, including a persistent pulling sensation, skin irritation, and a foreign body sensation at the fixation site [32]. Several patients declined reapplication of the tape once its adhesion had failed. In contrast, those patients who were managed without external fixation reported greater comfort and valued the ability to self-adjust the catheter. As Blumenthal-Barby [33] noted, patient values and preferences are integral to defining care value. A nursing practice in which comfort is systematically reduced and patient preference are disregarded—even if routine—cannot be considered to be high-value [9].

### Contextualizing the results within the existing evidence base

Our results appear to contradict some existing guidelines that advocate for secure external fixation [11,13]. This discrepancy can be explained by fundamental differences among patient populations and clinical scenarios. Prior research and recommendations have often concerned critically ill, immobilized, or long-term catheterized patients [8,30], who are inherently at a greater risk of catheter dislodgment. Our study specifically targeted a distinct group: post-TURP patients with short-term catheterization, normal consciousness, and high levels of compliance undergoing planned early mobilization. For these patients, catheter security is adequately maintained by the balloon, patient cooperation, and the innovative use of a modified gown, thereby rendering external adhesive fixation unnecessary. This approach is not only safe but also in alignment with ERAS objectives [10].

The absence of a significant difference in CAUTI rates between groups is consistent with evidence used to identify short catheter duration as the foremost modifiable risk factor for infection [12,34]. The standardized patient education on infection prevention practices, which were provided to both groups, likely mitigated any potential risk differential arising from the fixation method itself.

### Research and practical implications

This study addresses a notable gap in the current literature concerning the catalysis of low-value care. Neither China's nursing low-value item list [15] nor international “choosing wise” campaigns [16–18] have explicitly identified routine external catheter fixation as a low-value practice. As Davidson et al. [24] emphasized, the deimplementation of such practices requires robust evidence. Our RCT provides precise evidence indicating that “routine external body-surface fixation of urinary catheters in ambulatory post-TURP patients” should be considered for inclusion in such lists.

From a clinical perspective, our findings support a paradigm shift in postoperative catheter management for this population. Adopting a nonexternal fixation protocol—which entails the use of a modified gown, systematic patient education and the elimination of routine adhesive tape application—can reduce iatrogenic injury, lower costs, decrease the nursing workload, and improve patient comfort, all without compromising safety. Thus, this change aligns perfectly with the goals of ERAS and those of value-based nursing care.

## Limitations

The conclusions of this study should be interpreted in light of its limitations. First, as a single-center trial, the generalizability of the findings across other institutions or healthcare systems may be limited. Second, the study cohort consisted of relatively healthy and compliant patients, so the results may not extend to those with cognitive impairment, agitation, or those who require prolonged catheterization. Third, our economic analysis was confined to direct costs (materials and nursing time), whereas the indirect costs associated with potential complications were not captured. Fourth, patient comfort was assessed qualitatively; future research would benefit from the use of validated, patient-reported outcome measures [9]. Finally, the follow-up period was limited to the catheterization timeframe, and while UCR-MPI is typically an acute injury [8], longer-term outcomes were not assessed.

## Conclusion

In summary, for patients mobilizing early after TURP, routine external fixation of the urinary catheter to the body surface satisfies all of the established attributes of low-value care. Such fixation offers no clinical advantage over nonfixation, increases the risk of meatal pressure injury, incurs unnecessary costs, and diminishes patient comfort. The nonexternal fixation strategy, as supported by simple garment modification and structured education, emerges as a safe, effective, patient-centered, and resource-efficient alternative. This study provides high-quality RCT evidence to inform clinical guidelines and support the deimplementation of a common yet low-value nursing practice. Further multicenter studies are encouraged to confirm these findings in diverse settings and patient groups.

## Supporting information

S1 File CONSORT-checklist. (DOCX)

S2 File Original protocol. (PDF)

S3 File Data. (PDF)
