# Supplementary material for: Catheter body-surface fixation after transurethral prostate resection: A low-value nursing practice as evidenced in a randomized controlled trial
Source: PLoS One. 2026 Jun 4;21(6):e0350800. doi: 10.1371/journal.pone.0350800 (PMC13235868; doi:10.1371/journal.pone.0350800)
Supplement: S2 File — (PDF) [file pone.0350800.s002.pdf]

项目编号：

# 青岛市市立医院

# 临床研究项目立项文件

项目名称：“放养式”固定留置导尿管在经尿道前列腺切除术术后患者中的应用研究

研究科室：泌尿外科

主要研究者：董海静

联系方式：18661678235

合作单位：

研究期限：2024.6-2025.5

学术编号：XS202405034

伦理编号：2024-LW-047（快）

二0二三年制

青岛市市立医院

# 项目计划书（括号内容删除，注：以下内容与研究方案模板基本一致）

| | | | |
|---|---|---|---|
| **课题名称** | “放养式”固定留置导尿管在经尿道前列腺切除术术后患者中的应用研究 | | |
| **课题负责人** | 董海静 | **承担单位** | 青岛市市立医院 |
| **研究性质** | 干预性研究 | **研究领域** | 泌尿外科 |
| **所属学科** | 护理 | **起止日期** | 2024.6-2025.5 |

**1、 研究本课题的科学依据**

1.1 研究背景

良性前列腺增生（Benign Prostatic Hyperplasia，BPH）是中老年男性最常见的疾病之一，据报道，50 岁以上男性发病率大于 50%，80 岁以上男性发病率达到 90%[1-3]。良性前列腺增生常导致膀胱出口梗阻、排尿困难等下尿路阻塞症状，最终引起膀胱和肾脏损害，成为影响中老年男性健康的重要疾病之一[4]。经尿道前列腺切除术（Transurethral Resection of Prostate，TURP）是解除 BPH 导致膀胱流出道梗阻外科治疗的“金标准”[5]，术后常需留置导尿管以引流膀胱内尿液，减轻尿道水肿和疼痛，并早期识别术后出血和尿潴留等并发症。

1.2 研究必要性

目前多项研究指出，应常规对留置导尿管进行外固定，以防止导尿管反复移动，导致膀胱括约肌松弛、膀胱痉挛，或导致显著的尿道、膀胱颈机械性损伤，引起导尿管相关性感染[6-8]。但对外固定的必要性研究多开展于重症监护病房或神经内科病房，此类病人因病情需要留置导尿时间较长，且易发生谵妄等意识状态的变化导致自行拔管，因此需要妥善固定留置导尿管。但基于加速康复外科理念经尿道前列腺切除术后 3 天即可拔管[9],术后留置尿管时间较短，是否有必要进行外固定尿管值得探讨。

1.3 参考文献

[1] Turgut Ö, Erbagcı A, Bayrak O, Seckiner I, Erturhan S, Sen H, et al. Correlation of postoperative outcomes according to the amount of prostatic tissue removed in patients undergoing transurethral resection of the prostate. Cureus. 2023;15(1):e34451.

[2] 李胜,曾宪涛,李晓东,等.良性前列腺增生临床实践指南的质量评价[J].中国循证医学杂

志.2018,18(01):74-80.
[3] Elterman D, Aubé-Peterkin M, Evans H, et al. UPDATE - Canadian Urological Association guideline: male lower urinary tract symptoms/benign prostatic hyperplasia. Can Urol Assoc J. 2022,16(8):245-256
[4] Ichiyanagi O, Ishigooka M, Hashimoto T, et al. The American Urological Association Symptom Index: early postoperative evaluation of irritative and obstructive symptoms due to benign prostatic hyperplasia. Int Urol Nephrol. 1997;29(4):441-7.
[5] Karkhanei B, Musavi-Bahar SH, Bayat M, et al. Safety and efficacy of intraoperative administration of intravenous tranexamic acid in transurethral resection of prostate: A double-blind, randomised, placebo-controlled trial. J Clinic Urol. 2019,13(2):205141581985588.
[6]许宾.不同留置导尿管外固定方法对神经内科男性患者短期留置导尿的影响[J]. 中国现代药物应用. 2022;16(04):47-9.
[7]王得莹.留置导尿管外固定方法对男性患者短期留置导尿的影响[J].中国医疗器械信息,2019;25(18):95-6.
[8] 苏红侠,陈超豪,黄盈,等.经尿道前列腺电切术后气囊导尿管固定方式比较[J].浙江实用医学,2019,24(03):227-229.
[9] 陈真飞.导管护理对导管相关性尿路感染的干预效果评价[J].当代医学.2018,24(07):174-175.
[10]马铮铮,钮美娥,王卫珍,等.持续膀胱冲洗引流液颜色评估工具一致性检验的研究[J].中国护理管理,2021;21(02):207-11.
[11] Shafshak TS, Elnemr R. The visual analogue scale versus numerical rating scale in m-easuring pain severity and predicting disability in low back pain. J Clin Rheumatol. 2021;27(7):282-5.
[12]龚若,冷虹瑶,余鸿凡,等.不同评估方法在肺切除术患者围手术期疼痛测量中的心理测量学特征[J].陆军军医大学学报.2023;45(20):2132-40.
[13] 李真,蔡宁,马行军,等.老年膝关节置换患者最优麻醉方案选择及对膝关节功能、术后疼痛的影响[J].中国老年学杂志,2023;43(21):5202-5.

**1.2 实验目的**

探讨 TURP 术后留置导尿管外固定的必要性。

**2、研究内容和预期成果**

**2.1 研究内容**

选取符合纳入标准的 TURP 术后留置导尿管的患者分为对照组和实验组，两组采取相同的留置导尿管护理措施，对照组行留置导尿管外固定，试验组采用“放养式”固定，观察两组患者留置导尿管期间有无尿管脱落、血尿、尿道疼痛、尿道口压力性损伤发生情况及拔除导尿管后尿培养和前列腺症状情况。

2.2 研究对象、样本量及明确的样本量计算方式

纳入标准：①符合国家《良性前列腺增生症诊疗指南》所列手术指征的良性前列腺增生（BPH）患者； ②术前尿培养结果阴性；③所有手术均由同一医疗团队实施以确保手术技术标准化；④无肢体活动障碍或沟通障碍；⑤术后病理学确诊前列腺组织增生；⑥所有患者知情同意。排除标准：①术后留置导尿管时间＜1 天或>3 天；②术前有凝血功能障碍、穿刺部位感染、急性或慢性疼痛、药物成瘾的患者；③合并其他泌尿系相关疾病，既往有长期留置导尿史；④医用粘贴过敏者。

2.3 入组患者数量与计算依据

在患者入组前的研究设计阶段计算样本量。根据主要结局（UCR-MPI，术后第 3 天≥I 级）计算样本量。基线发病率（10.7%，13/121）来自我们中心的回顾性 TURP 患者数据。我们假设非外固定将使这一比例降至 1.0%。使用 $\alpha=0.05$（双侧，$Z_{\alpha/2}=1.96$）和功率=80%（$Z_{\beta}=0.84$）的两个独立比例公式，每组需要 88 名患者。考虑到 10%的退出率，我们每组招募了 105 名患者（共 210 名），最后 208 名患者提供了足够的能量。

$N=(Z_{\alpha/2}+Z_{\beta})^2\times[P0(1-P0)+P1(1-P1)]/(P0-P1)^2$

参数：P0=0.107（基线），P1=0.01（假设）。选取 2024 年 6 月~2025 年 5 月于青岛市市立医院东院区泌尿外科接受 TURP 手术术后留置导尿管的患者 210 例。

2.4 分组方法

随机分为实验组和对照组，两组患者均由同一组医师完成手术。使用计算机生成的随机序列，按 1:1 比例将患者分配至两组。分配序列由不参与手术和术后直接护理的研究护士密封保存，在患者返回病房后拆封并执行分组。

盲法： 由于干预性质，对患者和实施干预的护士无法设盲。但对结局评估者（研究护

士） 和数据分析人员实施盲法。负责评估尿道口黏膜压力性损伤、血尿颜色等的护士不知晓患者的分组情况。

2.5 干预措施

两组患者的集尿袋置于膀胱以下位置并及时排空；每日两次清洁消毒会阴部、尿道口和导尿管；术后 6 小时下床活动时穿着宽松避免衣物对导尿管的牵引；术后生理盐水持续膀胱冲洗直至为淡黄色，可拔除；拔除导尿管时抽尽气囊后再打入 0.3~0.5ml 谁，与腹壁呈 60 度角时拔除导尿管。

对照组患者采用传统固定法，统一采用 3M 医用弹力胶布高举平台将尿管固定于大腿内侧，保持尿道口与皮肤固定点长度适中。患者卧位时，尿管从大腿上方走行，活动时将导尿管悬挂于腰带上，尽量减少尿袋的重力；

实验组患者采用“放养式”固定，留置导尿管后不再患者体表进行二次固定，患者穿着特制病员裤（在双侧裤腿膝盖上方约 5-10cm 处设有隐蔽开口）。导尿管及引流管从此开口穿出，尿袋固定于裤腿。此设计使导尿管自然悬垂，减少对外生殖器的牵拉，病人可根据主观感受调节尿管走向及松动度,并便于活动时观察尿量。卧床时，尿袋常规置于近侧床栏。

2.6 主要与次要研究指标

（1）术后疾病恢复指标：

①非计划性拔管率：计算公式为：（该组非计划性拔管事件总数 / 该组总导管日）× 1000‰。

②留置尿管期间患者血尿情况：采用尿液颜色比色卡获取患者手术当日下床活动时、术后第 1～2 日晨起站立的尿液颜色。比色卡共有 6 个色阶，对应 6 个血液浓度（红细胞浓度 8%、4%、2%、1%、0.5%、0.125%）[10]。在本次研究获取尿色时，将引流液挤入透明软引流管中进行比色，确定引流液颜色所属色阶；若比色结果介于两色阶之间，则以高色阶作为颜色参考。

③尿道口黏膜压力性损伤情况：观察尿道外口有无红肿，黏膜有无破溃；

④拔除导尿管后尿培养。

⑤人力成本（护理工作量）： 记录与导管固定相关问题（如胶带脱落、重新固定、处理卷边等）相关的护理干预频次。

⑥耗材成本： 对照组计算 3M™医用弹力胶带（标准化为 5cm×7cm/条）的使用成本。实验组无此项耗材成本。

（2）术后患者感受指标：

①疼痛情况：采用数字疼痛评估法（numerical rating scale，NRS）评估患者手术当日下床活动时、术后第1～2日活动状态及平静休息状态的疼痛评分。NRS采用0～10的数值来衡量患者的疼痛程度的评价工具，其中，0代表无疼痛，10代表剧痛[11-13]。

②前列腺症状：采用国际前列腺症状评分表（IPSS）对拔除导尿管后的患者的前列腺症状进行评分。通过是否存在有排尿不尽感、两次排尿短于2小时、经常间断性排尿、经常憋尿困难、经常尿线变细、经常排尿费力、夜尿次数7个问题进行评价，每个问题得分0~5分。分数越高，前列腺症状越严重[14,15]。在本次研究中，入院时，获取两组患者前列腺症状的基线资料，患者拔出留置导尿管后第1天，再次评价患者的前列腺症状评分情况。

2.7 安全性与风险分析

无风险。

2.8 统计学方法

应用Epidata3.0软件双人录入数据，SPSS 21.0软件进行统计学分析，数据描述性分析，计数资料采用频数、百分比描述，计量资料采用均数±标准差（±S）进行描述性分析；组间比较，计量资料采用独立样本t检验，计数资料采用卡方检验。P＜0.05表示差异具有统计学意义。

**2.2 预期成果**

1.发表论文1篇；

2.指导更新留置导尿管护理的规范与流程。

**2.3 研究进度（以年或半年为单位）**

2024.3-2024.6 确定课题方向、科研方案，编制相关调查量表；

2024.6-2025.5 临床数据收集；

2025.5 分析数据，撰写论文。

**3、现已具备的条件（含可行性分析）**

（1）技术可行性：本研究采用的方法均为成熟且广泛应用的医学研究方法，可实施。

（2）数据可行性：青岛市市立医院东院区泌尿外科每月行TURP手术患者每周约20例，通过收集和分析临床病例数据，可以获得充足的研究样本和数据支持，确保研究的可靠性和有效性。

（3）伦理可行性：本研究符合医学伦理规范，保障患者的权益和安全，同时确保研究结果的公正性和客观性。

**4、实验涉及的伦理问题**

本研究严格遵守《赫尔辛基宣言》原则。所有参与者在入组前均需充分了解研究目的、过程、潜在风险和获益，并知情同意。参与者有权在任何时候无条件退出研究，且不影响其后续治疗。本研究需从医疗系统获取患者信息，在整个研究过程中均未透漏患者身份信息。研究数据将被去标识化处理，仅用于本研究目的，并严格保密。

**5、课题或项目承担单位、参加单位及分工**

承担单位：青岛市市立医院东院区

分工：

董海静：研究设计、统筹安排

朱亚南：研究设计、数据收集、论文撰写

张欣红：数据收集、研究设计、论文撰写

王倩：数据收集、统计分析、论文撰写

贾会英：研究设计

赵改云：资料整理

**6、经费来源**

☐企业资助　公司名称：　资助金额：

☐基金　基金名称：　资助金额：

☑自筹

**7、经费预算**

材料费 5000 主要用于文献检索、材料印刷和课题总结

培训费 2000 参加学术会议学习费用等

论文投稿及发表 13000 发表研究论文 1-2 篇版面费用

**Project Number:**

# Qingdao Municipal Hospital
# Clinical Research Project Approval Document

**Project Name: Application Study of "Unrestricted Fixation" Indwelling Urinary Catheters in Postoperative Patients Undergoing Transurethral Prostatectomy**

**Graduate School: Department of Urology**

**Principal Investigator: Dong Haijing**

**Contact Information: 18661678235**

**Collaborating Institution:**

**Research Period: June 2024–May 2025**

**Academic Number: XS202405034**

**Ethics Number: 2024-LW-047（快）**

Made in 2023

Qingdao Municipal Hospital

# Project Proposal (Content in parentheses deleted. Note: The following content is largely consistent with the research proposal template.)

| | | | |
|---|---|---|---|
| Project Title | Application Study of "Unrestricted Fixation" Indwelling Urinary Catheters in Postoperative Patients Undergoing Transurethral Prostatectomy | | |
| **Project Leader** | Dong Haijing | Implementing Entity | Qingdao Municipal Hospital |
| **Study Type** | Interventional Study | **Research Field** | Urology |
| **Discipline** | Nursing | **Duration** | June 2024 to May 2025 |
| 1、Scientific rationale for undertaking this research project | | | |

1.1 Research Background

Benign prostatic hyperplasia (BPH) ranks among the most prevalent conditions affecting middle-aged and elderly men. Reports indicate a prevalence exceeding 50% in males over 50 years of age, rising to 90% in those aged 80 and above [1-3]. BPH frequently causes lower urinary tract obstruction symptoms such as bladder outlet obstruction and dysuria, ultimately leading to bladder and renal damage, making it a significant health concern for middle-aged and elderly men [4]. Transurethral resection of the prostate (TURP) is the gold standard surgical treatment for relieving bladder outlet obstruction caused by BPH [5]. Postoperative indwelling urinary catheters are frequently required to drain urine from the bladder, alleviate urethral oedema and pain, and enable early detection of complications such as postoperative haemorrhage and urinary retention.

1.2 Research Rationale

Multiple studies currently indicate that external fixation of indwelling urinary catheters should be routinely implemented to prevent recurrent catheter displacement. Such displacement may lead to bladder sphincter relaxation, bladder spasm, or significant mechanical injury to the urethra and bladder neck, thereby causing catheter-associated infections [6-8]. However, studies on the necessity of external fixation have predominantly been conducted in intensive care units or neurology wards. Patients in these settings often require prolonged catheterisation due to their

medical condition and are prone to altered mental states such as delirium, which may lead to self-catheter removal. Consequently, secure fixation of indwelling catheters is essential. However, based on the principles of enhanced recovery after surgery, urinary catheters may be removed as early as three days post-transurethral resection of the prostate [9]. Given the relatively brief postoperative catheterisation period, the necessity of external catheter fixation warrants further consideration.

## 1.3References


[1] Turgut Ö, Erbagcı A, Bayrak O, Seckiner I, Erturhan S, Sen H, et al. Correlation of postoperative outcomes according to the amount of prostatic tissue removed in patients undergoing transurethral resection of the prostate. Cureus. 2023;15(1):e34451.

[2] Li S, Zeng XT, Li XD, et al. Quality Evaluation of Clinical Practice Guidelines for Benign Prostatic Hyperplasia [J]. Chinese Journal of Evidence-Based Medicine.2018,18(01):74-80.

[3] Elterman D, Aubé-Peterkin M, Evans H, et al. UPDATE - Canadian Urological Association guideline: male lower urinary tract symptoms/benign prostatic hyperplasia. Can Urol Assoc J. 2022,16(8):245-256.

[4] Ichiyanagi O, Ishigooka M, Hashimoto T, et al. The American Urological Association Symptom Index: early postoperative evaluation of irritative and obstructive symptoms due to benign prostatic hyperplasia. Int Urol Nephrol. 1997;29(4):441-7.

[5] Karkhanei B, Musavi-Bahar SH, Bayat M, et al. Safety and efficacy of intraoperative administration of intravenous tranexamic acid in transurethral resection of prostate: A double-blind, randomised, placebo-controlled trial. J Clinic Urol. 2019,13(2):205141581985588.

[6] Xu B. Effects of Different External Fixation Methods for Indwelling Urinary Catheters on Short-Term Catheterisation in Male Neurological Patients [J]. Chinese Journal of Modern Drug Application. 2022;16(04):47-9.

[7] Wang DY. The Effect of External Fixation Methods for Indwelling Urinary Catheters on Short-Term Catheterisation in Male Patients [J]. China Medical Devices Information.2019;25(18):95-6.

[8] Su HX, Chen CH, Huang Y, et al. Comparison of Fixation Methods for Foley Catheters Following Transurethral Resection of the Prostate [J]. Zhejiang Practical Medicine.2019,24(03):227-229.

[9]Chen ZF. Evaluation of the Effectiveness of Catheter Care in Preventing Catheter-Associated Urinary Tract Infections [J]. Contemporary Medicine.2018,24(07):174-175.
[10]Ma ZZ, Niu ME, Wang WZ, et al. A Study on the Consistency Test of the Colour Assessment Tool for Continuous Bladder Irrigation Drainage Fluid [J]. Chinese Journal of Nursing Management.2021;21(02):207-11.
[11] Shafshak TS, Elnemr R. The visual analogue scale versus numerical rating scale in m-easuring pain severity and predicting disability in low back pain. J Clin Rheumatol. 2021;27(7):282-5.
[12] Gong R, Leng HY, Yu HF, et al. Psychometric characteristics of different assessment methods in measuring perioperative pain in patients undergoing lung resection [J]. Journal of Army Medical University.2023;45(20):2132-40.
[13] Li Z, Cai N, Ma XJ, et al. Selection of Optimal Anaesthetic Regimens for Elderly Patients Undergoing Knee Arthroplasty and Their Effects on Knee Function and Postoperative Pain [J]. Chinese Journal of Geriatrics.2023;43(21):5202-5.

1.2 Objectives of the Study

To investigate the necessity of external fixation for indwelling urinary catheters following transurethral resection of the prostate (TURP).

**2. Research Content and Expected Outcomes**

2.1 Study Content

Patients meeting inclusion criteria who underwent transurethral resection of the prostate (TURP) with indwelling urinary catheters were divided into a control group and an experimental group. Both groups received identical catheter care protocols. The control group underwent external fixation of the indwelling catheter, while the experimental group employed a ‘free-flowing’ fixation method. The study observed the occurrence of catheter dislodgement, haematuria, urethral pain, and pressure injuries at the urethral orifice during catheterisation in both groups. Additionally, post-catheter removal outcomes including urine culture results and prostate-related symptoms were monitored.

2.2 Study Population, Sample Size, and Defined Sample Size Calculation Method

Inclusion Criteria: ① Patients with benign prostatic hyperplasia (BPH) meeting surgical

indications as outlined in the National Guidelines for the Diagnosis and Treatment of Benign Prostatic Hyperplasia; ② Negative preoperative urine culture results; ③ All procedures performed by the same surgical team to ensure standardised surgical technique; ④ No limb mobility impairment or communication difficulties; ⑤ Postoperative pathological confirmation of prostatic tissue hyperplasia; ⑥ Informed consent obtained from all patients. Exclusion Criteria: ① Postoperative indwelling catheterisation duration < 1 day or > 3 days; ② Preoperative coagulation disorders, puncture site infection, acute or chronic pain, or substance dependence; ③ Concurrent other urological conditions, history of prolonged indwelling catheterisation; ④ Allergy to medical adhesives.

2.3 Number of Patients to be Enrolled and Calculation Basis

Sample size was calculated during the study design phase before patient enrollment.Sample size was calculated based on the primary outcome (UCR-MPI, grade ≥ I by postoperative day 3). Baseline incidence (10.7%, 13/121) was derived from our center's retrospective TURP patient data. We hypothesized non-external fixation would reduce this to 1.0%.

Using the two independent proportions formula with $\alpha=0.05$ (two-sided, $Z_{\alpha/2}=1.96$) and power=80% ($Z_{\beta}=0.84$), 88 patients per group were required. Accounting for 10% dropout, we enrolled 105 patients per group (total 210), with the final 208 patients providing sufficient power.

$$N = (Z_{\alpha/2} + Z_{\beta})^2 \times [P0(1-P0) + P1(1-P1)] / (P0 - P1)^2$$

Parameters: P0=0.107 (baseline), P1=0.01 (hypothesized). The study enrolled 210 patients who underwent transurethral resection of the prostate (TURP) surgery and required indwelling urinary catheters in the Department of Urology at Qingdao Municipal Hospital East Campus between June 2024 and May 2025.

2.4 Grouping Method

Patients were randomly assigned to an experimental group and a control group, with all procedures performed by the same surgical team. A computer-generated random sequence allocated patients to the two groups in a 1:1 ratio. The allocation sequence was sealed by a research nurse not involved in surgery or immediate postoperative care, and opened upon the patient's return to the ward for grouping implementation.

Blinding: Due to the nature of the intervention, blinding was not feasible for patients or the

nurses administering the intervention. However, blinding was implemented for the outcome assessors (research nurses) and data analysts. Nurses responsible for evaluating urethral orifice mucosal pressure injuries, haematuria colour, and other parameters were unaware of patient groupings.

2.5 Interventions

In both groups, urine collection bags were positioned below the bladder level and emptied promptly; perineal, urethral orifice and catheter areas were cleaned and disinfected twice daily; patients were encouraged to ambulate six hours postoperatively wearing loose clothing to prevent traction on the catheter; continuous saline bladder irrigation was maintained until urine appeared pale yellow, at which point the catheter could be removed. When removing the catheter, deflate the balloon completely before injecting 0.3 – 0.5 ml of saline. Withdraw the catheter at a 60-degree angle to the abdominal wall.

The control group employed conventional fixation: 3M medical elastic adhesive tape was uniformly applied to secure the catheter high on the inner thigh, maintaining an appropriate length between the urethral orifice and the skin fixation point. When the patient is recumbent, the catheter runs over the thigh. During ambulation, the catheter is suspended from the waistband to minimise the gravitational pull of the urine bag.

Patients in the experimental group underwent a ‘free-range’ fixation method. Following catheter insertion, no secondary fixation was applied to the patient's body surface. Patients wore specially designed hospital trousers (featuring concealed openings approximately 5 – 10 cm above the knees on both legs). The catheter and drainage tube exit through these openings, with the urine bag secured to the trouser leg. This design allows the catheter to hang naturally, reducing traction on the external genitalia. Patients can adjust the catheter's trajectory and slackness according to personal preference, facilitating observation of urine output during movement. When bedridden, the urine bag is routinely positioned near the bed rail.

2.6 Primary and Secondary Research Indicators

(1) Postoperative Recovery Indicators:

① Unplanned Extubation Rate: Calculated as: (Total number of unplanned extubation events in this group / Total catheter days in this group) × 1000‰.

② Haematuria during catheterisation: Urine colour was assessed using a colour chart during ambulation on the day of surgery and upon rising on postoperative days 1 – 2. The chart comprises six colour grades corresponding to six haemoconcentration levels (8%, 4%, 2%, 1%, 0.5%, 0.125% red blood cell concentration) [10]. For this study, drainage fluid was squeezed into a transparent flexible drainage tube for colour comparison to determine the corresponding scale level. Where the result fell between two scale levels, the higher level was used as the reference colour.

③ Mucosal pressure injury at the urethral orifice: Observe for redness and swelling at the external urethral orifice, and assess for mucosal ulceration;

④ Urine culture following catheter removal.

⑤ Labour costs (nursing workload): Record the frequency of nursing interventions related to catheter fixation issues (e.g., tape detachment, re-fixation, managing rolled edges).

⑥ Consumables cost: Calculate the usage cost of 3M™ Medical Elastic Tape (standardised as 5cm × 7cm per strip) for the control group. The experimental group incurs no such consumables cost.

(2) Postoperative patient-reported outcomes:

① Pain assessment: The Numerical Rating Scale (NRS) was employed to evaluate pain scores during ambulation on the day of surgery, as well as during activity and at rest on postoperative days 1 – 2. The NRS is a 0 – 10 numerical tool measuring pain intensity, where 0 denotes no pain and 10 signifies severe pain [11 – 13].

② Prostate Symptoms: The International Prostate Symptom Score (IPSS) was employed to evaluate prostate symptoms in patients following catheter removal. Assessment comprised seven questions: presence of a sensation of incomplete bladder emptying, urination less than two hours apart, frequent interrupted urination, frequent difficulty initiating urination, frequent weak urine stream, frequent straining during urination, and nocturia. Each question scored 0 – 5 points. Higher scores indicate more severe prostate symptoms [14,15]. In this study, baseline prostate symptom data were collected for both groups upon admission. Prostate symptom scores were reassessed on the first day following catheter removal.

2.7 Safety and Risk Analysis

No risk.

2.8 Statistical Methods

Data were entered by two operators using Epidata 3.0 software and analysed statistically with SPSS 21.0 software. Descriptive analysis was performed: categorical data were presented as frequencies and percentages, while continuous data were described using mean ± standard deviation ( ± S). Intergroup comparisons employed independent samples t-tests for continuous variables and chi-square tests for categorical variables. A P value < 0.05 was considered statistically significant.

2.2 Expected Outcomes

1. Publication of one research paper;

2. Guidance on updating standards and procedures for indwelling urinary catheter care.

2.3 Research Timeline (by year or half-year)

March – June 2024: Define research direction and methodology; develop relevant survey instruments.

June 2024 – May 2025: Collect clinical data.

May 2025: Analyse data and draft research paper.

**3. Conditions currently in place (including feasibility analysis)**

(1) Technical feasibility: All methodologies employed in this study are established and widely adopted medical research approaches, rendering implementation feasible.

(2) Data Feasibility: The Urology Department at Qingdao Municipal Hospital East Campus performs approximately 20 TURP procedures weekly. By collecting and analysing clinical case data, sufficient research samples and data support can be obtained, ensuring the reliability and validity of the study.

(3) Ethical Feasibility: This study complies with medical ethical standards, safeguarding patient rights and safety while ensuring the impartiality and objectivity of the research findings.

**4. Ethical issues involved in the experiment**

This study strictly adheres to the principles of the Declaration of Helsinki. All participants must fully understand the purpose, procedures, potential risks, and benefits of the research prior to enrolment and provide informed consent. Participants retain the right to withdraw from the study at any time without condition, and this will not affect their subsequent treatment. Patient information required for this study is obtained from healthcare systems, and no patient identifiers were disclosed throughout the research process.Research data will be de-identified and used solely for the purposes of this study, with strict confidentiality maintained.

**5. Project or Programme Lead Organisation, Participating Organisations and Division of Responsibilities**

Undertaking Unit: Qingdao Municipal Hospital East Campus

Division of Responsibilities:

Dong Haijing: Research design, overall coordination

Zhu Yanan: Research design, data collection, manuscript drafting

Zhang Xinhong: Data collection, research design, manuscript drafting

Wang Qian: Data collection, statistical analysis, manuscript drafting

Jia Huiying: Research design

Zhao Gaiyun: Data collation

**6. Funding Sources**

☐Corporate Funding Company Name： Funding Amount：

☐Amount Funding Source： Funding Amount：

☑Self-funded

**7. Budget**

Materials Fee ￥5000 Primarily allocated towards literature searches, material printing and project summaries

Training Fee ￥2000 Covering attendance at academic conferences and related

learning expenses

Paper Submission and Publication ￥13000 Publication fees for 1-2 research papers
