# Supplementary material for: Catheter body-surface fixation after transurethral prostate resection: A low-value nursing practice as evidenced in a randomized controlled trial
Source: PLoS One. 2026 Jun 4;21(6):e0350800. doi: 10.1371/journal.pone.0350800 (PMC13235868; doi:10.1371/journal.pone.0350800)
Supplement: S3 File — (PDF) [file pone.0350800.s003.pdf]

Category	age	BMI	religious	Education	Smoking	Preoperative pros
1	83	24.69	1	4	1	41.24
1	66	34.57	1	3	1	65.36
1	61	29.76	1	2	2	88.79
1	86	24.49	1	2	1	106.2
1	75	25	1	1	1	62.19
1	64	27.44	1	3	1	22.28
1	71	24.22	1	2	1	27.66
1	67	27.04	2	2	2	37.44
1	63	26.56	1	2	1	4.68
1	69	20.37	1	2	2	28.29
1	80	24.61	1	2	1	20.52
1	64	27.44	1	3	1	22.28
1	69	26.79	1	3	1	47
1	79	24.69	1	3	1	46.19
1	75	25.06	1	3	1	41.24
1	63	29.05	1	2	1	40.42
1	63	29.38	1	2	1	3.12
1	59	25.14	1	2	2	53.52
1	63	26.56	1	2	1	4.68
1	72	23.94	1	3	1	41.24
1	63	29.05	1	2	1	40.42
1	71	28.08	1	2	2	105.28
1	73	25.26	1	4	1	55.16
1	67	27.04	2	2	2	37.44
1	71	28.37	1	2	1	74.98
1	61	29.76	1	2	2	88.79
1	59	24.8	1	1	1	86.51
1	81	22.64	1	4	1	86.51
1	75	25.06	1	3	1	86.51
1	71	28.37	1	2	2	23.13
1	59	24.8	1	1	1	86.51
1	86	24.49	1	2	1	106.2
1	76	22.41	1	2	1	78.3
1	62	19.03	1	1	1	73.17
1	66	23.18	1	2	2	22.63
1	52	22.03	1	2	1	47
1	76	25.95	1	2	1	95
1	58	22.49	1	2	2	83
1	80	20.43	1	4	1	82
1	68	24.8	1	4	2	92
1	70	24.49	1	3	1	117
1	74	26.57	1	2	1	80
1	70	22.49	1	2	2	70
1	69	25.21	1	1	1	74.98
1	80	29.38	1	1	1	52
1	61	26.3	1	2	1	90
1	80	28.37	1	3	1	32
1	71	29.41	1	2	1	104
1	65	21.88	1	2	1	132
1	68	24.16	1	4	1	92
1	85	26.64	1	1	1	116
1	62	27.74	1	2	1	116
1	63	21.22	1	2	2	37
1	88	22.6	1	2	1	180
1	69	23.44	1	1	3	70

1	78	36.33	1	3	1	73
1	53	29.39	1	2	1	102
1	60	26.57	1	2	2	91
1	70	27.13	1	3	1	49
1	78	21.48	1	1	3	70
1	73	24.16	1	3	1	65
1	79	24.22	1	3	1	100
1	67	19.72	1	2	1	78
1	63	24.39	1	4	1	240
1	81	20.76	1	1	1	72
1	71	27.13	1	3	1	77
1	70	28.72	1	1	2	90
1	72	18.11	1	2	1	66
1	59	29.07	1	3	1	165
1	65	21.71	1	2	1	76
1	70	23.67	1	3	3	73.17
1	71	21.48	1	2	1	141
1	78	23.41	1	4	2	115
1	79	20.83	1	3	1	70
1	75	30.93	1	3	3	65
1	77	18.37	1	1	2	67
1	77	18.37	1	1	1	68
1	79	26.42	1	4	1	77
1	83	25.08	1	4	2	26
1	66	21.56	1	3	1	151
1	63	22.04	1	3	1	70
1	80	24.21	1	4	1	124
1	87	22.49	1	1	1	62
1	77	19.03	1	1	1	41
1	73	26.89	1	2	1	42
1	74	25.1	1	3	1	122
1	66	24.22	1	1	1	52
1	68	29.05	1	2	1	36
1	69	22.49	1	1	1	42
1	66	27.76	1	3	1	33
1	74	15.79	1	1	1	32
1	80	21.26	1	3	1	112
1	69	36.3	1	3	1	28
1	88	18.94	1	2	2	34
1	75	22.86	1	4	1	70
1	62	27.16	1	2	2	40
1	70	23.99	1	2	1	74
1	80	24.8	1	2	2	74
1	75	25.06	1	3	1	140
1	54	21.97	1	3	2	60
1	62	31.24	1	3	1	26
1	76	27.68	1	3	1	150
1	74	25.65	1	2	1	123
1	73	22.6	1	3	1	63
1	54	21.97	1	4	1	115
2	83	24.69	1	4	1	41.24
2	62	24.57	1	4	1	87.05
2	81	22.64	1	4	1	41.24
2	67	25.71	1	2	1	45.65
2	71	28.08	1	2	2	105.28
2	83	24.69	1	4	1	41.24

2	71	24.22	1	2	1	27.66
2	65	19.49	1	2	2	3.12
2	62	24.57	1	4	1	87.05
2	71	23.39	1	4	1	40.74
2	70	22.86	1	1	1	86.51
2	75	25	1	1	1	62.19
2	74	26.93	1	2	1	111.65
2	65	19.49	1	2	2	3.12
2	80	24.61	1	2	1	20.52
2	69	20.37	1	2	2	28.29
2	62	19.03	1	1	1	73.17
2	66	34.57	1	3	1	65.36
2	73	25.26	1	4	1	55.16
2	67	26.73	1	3	1	88.79
2	65	25.06	1	2	1	32.45
2	75	30.93	1	3	3	66
2	71	28.37	1	2	2	23.13
2	63	29.38	1	2	1	0.1
2	75	24.22	1	4	1	36.5
2	76	22.41	1	2	1	78.3
2	66	23.18	1	2	2	22.63
2	79	24.69	1	3	1	46.19
2	67	26.73	1	3	1	88.79
2	59	25.14	1	2	2	53.52
2	71	28.37	1	2	1	74.98
2	72	23.94	1	3	1	86.51
2	65	25.06	1	2	1	32.45
2	75	30.93	1	3	3	66
2	74	26.93	1	2	1	111.65
2	90	17.3	1	1	2	100
2	69	24.82	1	2	1	131
2	64	25.61	1	3	1	88
2	65	29.39	1	1	1	78
2	70	26.3	1	3	2	79
2	67	28.52	1	2	1	47
2	65	27.88	1	3	2	118
2	74	28.41	1	4	1	143
2	60	26.12	1	3	1	81
2	70	25.35	1	2	3	58
2	64	28.4	1	3	1	112
2	78	26.45	1	3	1	72
2	78	22.09	1	2	1	26
2	85	22	1	3	1	68
2	59	28.34	1	2	1	78
2	80	23.99	1	2	1	204
2	77	27.04	1	4	1	145
2	61	25.18	1	4	1	40
2	67	22.84	1	4	1	70
2	78	19.2	1	1	1	189
2	75	22.92	1	3	2	109
2	73	21.22	1	4	1	96
2	71	15.57	1	2	1	157
2	71	25.25	1	4	1	74.98
2	71	23.08	1	4	1	72
2	80	28.03	1	1	1	40
2	68	22.92	1	4	2	49

2	85	25.53	1	3	1	83
2	64	22.02	1	4	1	70
2	66	21.38	1	3	1	54
2	80	24.57	1	3	1	223
2	65	26.64	1	3	1	93
2	73	23.15	1	2	2	50
2	64	25.88	1	4	1	26
2	71	28.34	1	3	3	189
2	72	26.12	1	2	2	50
2	84	23.39	1	3	2	73.17
2	87	19.59	1	1	2	70
2	86	22.86	1	4	1	140
2	68	27.34	1	2	1	67
2	77	29.38	1	3	1	39
2	70	20.76	1	2	1	69
2	66	24.8	1	3	1	80
2	65	25.54	1	4	1	56
2	73	27.25	1	4	1	43
2	70	25.35	1	3	1	69
2	67	22.65	1	2	2	52
2	70	24.21	1	2	3	24
2	83	23.92	1	3	1	98
2	86	22.49	1	3	1	80
2	76	27.68	1	2	1	62
2	75	30.1	1	2	1	65
2	85	24.69	1	4	1	80
2	71	25.95	1	3	1	76
2	77	22.49	1	2	1	60
2	76	18.14	1	1	1	47
2	74	23.31	1	4	1	72
2	64	22.77	1	2	2	70
2	70	26.88	1	4	1	61
2	86	28.4	1	1	1	78
2	69	23.99	1	2	1	184
2	69	22.32	1	3	1	65
2	74	25.65	1	2	1	78
2	63	23.52	1	2	2	30
2	80	29.41	1	1	1	50
2	69	23.99	1	2	1	140
2	68	23.52	1	2	2	40
2	77	23.19	1	4	2	117

Preoperative PSA	Preoperative resid	Number of ty	Anaesthesia	Length of surg
1.65	4.1	3	3	75
12.08	15	1	3	30
3.19	42	0	3	75
2.38	50	0	3	75
1.52	42	2	3	100
1.75	50	0	3	120
3.5	8	0	2	90
4.62	12	0	1	72
0.65	42	2	3	40
2.81	90	1	3	120
3.28	50	1	3	50
0.91	13.5	3	3	130
1.46	12	2	3	100
5.61	42	5	3	95
6.8	89.5	2	3	60
2.51	42	0	3	45
6.8	13.5	2	3	55
1.82	10	1	3	180
1.04	76	1	3	80
94.35	117	2	3	50
5.61	271	1	3	110
2.65	42	1	2	175
40.47	207	1	2	100
3.22	117	1	2	110
4.87	90	2	1	90
36.52	89.5	0	1	60
7.86	90	1	1	90
7.15	42	2	3	30
3.66	42	4	3	120
42.84	90	2	3	130
6.02	50	2	1	100
2.88	90	2	3	120
6.15	117	0	3	120
3.91	476	2	3	105
4.32	89.5	1	1	40
3.48	76	3	2	70
4.17	72	3	2	65
7.31	22	3	2	100
7	90	7	3	130
6.47	46	11	2	115
6.6	10	14	2	70
13.29	62	5	2	100
6.18	32	1	2	85
1.39	420	8	2	65
1.31	19	13	2	120
2.63	109	1	1	95
3.79	30	9	3	70
1.71	8	9	3	35
6.6	30	7	1	95
3.74	32	3	1	105
22.5	75	4	3	95
5.95	118	3	2	140
0.56	50	5	2	57
8.22	50	3	2	140
0.44	50	7	2	45

0.79	36	9	1	60
12.83	59	2	1	195
6.47	10	1	1	110
4.79	5	5	3	105
0.23	93	7	2	40
7.55	90	3	2	60
4.89	24	3	2	134
12.42	59	3	2	89
4.52	142	2	2	150
0.81	50	9	2	47
12.42	15	6	2	79
35.26	75	8	2	130
0.68	129	9	2	45
6.69	90	9	2	150
6.44	45	1	2	35
3.75	76	6	2	100
6.63	30	3	2	95
4.95	9	12	2	120
2.19	525	5	2	60
3.76	374	8	2	90
5.7	42	5	1	86
5.78	50	5	1	96
6.02	42	8	1	25
0.35	82	6	1	70
3.57	200	6	3	135
6.85	48	3	3	60
6.4	89.5	3	3	105
10.7	50	3	2	165
2.89	62	1	2	60
1.19	30	5	2	110
22.3	357	3	1	15
3.62	76	9	1	79
3.15	64	9	1	36
6.18	115	6	2	35
0	52	2	2	45
3.96	76	3	2	55
3.61	30	4	2	75
1.58	50	8	1	45
6.17	30	2	1	40
5.95	62	7	1	60
0.42	48	9	3	45
7.13	103	4	2	50
6.67	90	5	2	110
3.66	100	5	2	120
3.08	300	2	2	85
1.6	34	2	2	45
7.74	153	10	2	135
12.59	62	7	2	170
2.39	49.5	2	2	130
3.08	300	2	2	85
1.65	4.1	3	3	75
6.17	90	0	3	100
0.42	117	1	3	90
0.85	8	1	2	65
12.34	10	1	2	75
4.8	89.5	2	1	90

0.49	76	0	3	50
1	89.5	4	3	40
10.14	207	2	3	104
4.68	15	2	3	250
2.14	10	1	3	55
6.37	89.5	2	3	55
74.09	89.5	0	3	55
6.62	207	1	3	65
0.76	15	0	3	90
5.32	42	1	3	100
2.1	42	3	3	98
6.85	42	5	3	75
2.43	50	0	3	90
5.46	8	1	3	70
8.62	90	1	3	72
5.61	12	2	3	30
1.6	76	1	3	240
5.61	90	3	2	48
7.13	76	3	2	52
0.59	13.5	2	1	120
15.9	90	1	1	99
0.74	50	2	1	72
11.84	90	2	3	100
4.91	50	0	3	80
8.09	76	4	1	30
51.27	89.5	1	3	135
0.47	117	0	3	65
3.76	120	0	3	90
4.93	148	1	2	85
12.3	56	9	2	93
10.71	52	8	3	140
5.7	89.5	5	1	104
9.86	50	6	2	120
33.5	91	9	2	139
0.83	56	9	2	76
23.21	76	15	2	95
5.57	62	8	2	169
4.75	10	1	1	107
3.23	72	4	3	70
4.17	149	5	3	105
6.4	43	11	3	80
6.15	42	4	2	50
4.77	89.5	1	2	50
30.2	450	4	2	80
2.32	96	4	1	80
3.53	62	6	3	65
3.15	21	4	3	36
6.44	33	5	3	75
5.97	500	7	3	30
4.88	234	4	3	90
2.88	45	13	3	70
6.67	10	5	2	105
4.13	23	4	2	85
5.97	42	9	2	46
2.07	653	6	2	60
6.63	90	5	2	100

12.06	15	4	2	75
16.54	62	1	2	95
2.01	56	7	2	84
5.78	89.5	5	2	185
7.41	7.7	7	2	150
0.83	50	12	2	65
0.38	31	5	1	35
4.7	30	3	1	245
6.37	30	6	3	40
20.36	72	10	3	90
0.6	128	8	3	40
7.53	90	12	3	105
4.59	89.5	6	3	144
1.14	46	9	3	70
1.99	52	8	3	25
1.59	50	2	2	104
3.39	75	8	2	49
4.43	89.5	9	2	74
3.55	59	9	2	114
5	89.5	4	2	85
3.85	200	9	1	45
2.1	140	16	1	140
3.97	82	9	2	100
2.47	59	9	2	85
2.81	62	5	1	62
1.64	50	5	1	39
1.83	50	2	3	80
7	49.5	12	2	105
2.17	59	9	2	65
5.17	89.5	9	2	144
19.95	64	3	2	94
9.79	525	6	2	103
9.61	50	6	2	60
6.69	50	3	2	85
31.55	30	2	2	50
12.59	59	7	2	170
0.81	50	9	2	49
1.73	64	6	2	64
6.62	50	3	2	85
3.72	43	5	2	69
1.8	50	3	1	70

Postoperative bla	Postoperative analgesic and antispasmodic dr	Blood Concentration (n, %)
16	1	2
17	1	2
19	1	2
70	1	2
21.5	1	2
19	1	2
21	1	2
20	1	2
20	0	4
24	0	2
16	0	2
19	0	2
21	0	4
14	0	0
19.5	0	2
15	0	1
14	0	2
17.5	0	2
20	0	2
12	0	2
15	0	2
18	0	1
20	0	1
20	0	3
19	0	2
19	0	2
11.5	0	2
19.5	1	1
19.5	1	3
20	1	2
11.5	1	2
70	0	4
24	0	3
17.5	0	2
14	0	3
17	0	4
19	1	3
17	1	5
52	1	5
17	1	5
21	1	3
21	1	3
16	1	1
23	1	5
10	1	5
13	1	3
16	0	4
16	0	3
42	0	5
13	0	2
19	0	1
17	0	1
19	1	1
16	1	1
17	1	0

46	0	2
37	1	2
20.5	1	3
20	1	4
12	1	3
19	1	3
19	1	2
20	1	1
20.5	1	2
21	1	2
21	1	3
13	1	3
15	1	2
18	1	4
21	1	4
22	1	2
16	1	3
11	1	2
14	1	1
18	1	1
17	1	2
15.5	1	3
20	1	1
18	1	2
44.5	0	3
16	0	2
21	0	3
20	0	2
21	0	2
19	0	3
17	0	2
30	0	2
12	0	1
17	1	4
14	1	5
15	1	2
14	1	3
16	1	3
14	1	3
17	1	1
19	1	2
14	1	3
14	1	3
20	1	1
19	1	3
24	1	5
16.5	1	1
25	1	1
15	1	2
18	1	4
16	1	2
14	1	2
19.5	1	2
19	1	4
18	1	2
16	1	2

21	0	3
12	0	3
14	0	2
14	0	4
13	0	2
21.5	0	2
26	0	0
12	0	2
16	0	2
24	0	2
17.5	0	2
17	0	3
20	0	2
16	0	2
16	0	1
18	0	1
20	0	2
14	0	2
21	0	3
24	0	2
14	0	2
14	0	2
16	1	3
17.5	1	2
19	1	3
12	0	4
16	0	4
18	0	0
26	0	2
40	0	2
25	0	4
18	1	3
10	1	4
74	1	4
15	1	3
17	1	3
15	0	3
20	0	3
15	0	2
21	0	2
17	0	1
15	1	2
21	1	4
16	1	2
38	1	3
15	1	3
20	1	2
29	1	4
14	1	1
17	1	2
10	1	1
12	1	3
13	1	2
15	1	2
22	1	2
20	1	3

22	1	2
17	1	1
21	1	3
19	1	2
17	1	1
20	1	3
21	1	3
19	1	4
20	0	3
21	0	2
41	0	0
17	0	2
20	0	5
104	0	3
17	0	3
16	0	1
19	0	2
12	0	2
21	0	3
19	0	2
21	0	3
18	0	2
21	0	2
17	1	3
22	1	3
18	1	4
21.5	1	2
14	1	1
16	1	1
18	1	2
20	1	1
20	1	2
21	1	2
21	1	5
22	1	5
25	1	3
23	1	0
16	1	1
21	1	2
23	1	1
15	1	2

Blood Concentration (n, %)	Blood Concentration (n, %) - Seconc	Preoperative International Prc
1	1	26
1	1	22
1	1	23
2	3	31
2	1	19
2	2	22
2	2	25
2	2	23
2	1	22
1	1	22
1	1	20
1	1	17
3	1	22
2	1	27
2	2	31
1	1	26
2	2	27
1	1	25
2	2	23
2	2	22
1	1	26
1	1	22
1	1	26
3	2	20
2	2	20
1	1	23
2	2	17
1	1	27
2	1	31
2	1	30
2	2	17
3	3	31
2	2	23
2	1	30
2	1	22
3	3	23
1	1	23
3	3	19
3	2	23
4	1	27
5	2	31
1	1	27
1	3	20
4	2	31
3	2	35
2	2	25
2	1	20
5	2	31
3	2	32
1	1	23
1	1	27
2	1	30
1	1	30
1	1	23
1	1	30

1	1	19
1	1	21
1	1	23
2	1	18
1	1	21
2	2	16
1	1	24
1	2	23
1	1	23
1	1	22
2	1	20
1	1	23
1	3	23
1	1	31
1	1	23
1	1	18
1	1	23
1	1	27
1	1	15
1	1	30
1	1	20
1	1	27
1	1	19
1	1	22
2	1	23
2	1	30
1	1	16
2	1	23
1	1	18
3	1	23
1	1	31
5	3	18
1	1	24
2	2	23
2	2	20
1	1	14
1	1	32
1	1	18
2	1	18
1	1	27
2	2	18
1	3	20
1	1	27
1	1	23
1	1	23
2	2	20
1	1	27
1	1	19
1	1	20
1	2	25
1	1	26
1	1	24
2	1	27
2	1	20
1	1	21
2	2	26

1	1	22
1	1	22
1	1	21
2	1	21
1	1	22
1	1	19
0	0	15
1	1	22
1	1	19
2	2	22
2	1	30
2	2	22
2	1	26
2	1	25
2	1	23
1	1	28
1	3	30
1	1	27
1	1	20
1	1	23
2	2	22
2	2	27
1	1	25
2	1	25
3	3	20
3	1	22
3	1	23
2	1	28
1	1	18
3	4	23
3	2	29
1	1	23
2	2	15
2	2	22
2	1	21
2	2	23
2	2	23
5	3	18
1	1	23
1	1	29
1	1	22
1	1	23
2	1	22
1	1	23
1	1	21
1	1	19
1	1	23
1	3	21
1	1	22
1	1	18
2	1	26
2	1	25
1	1	19
1	1	21
1	1	19
1	1	18

2	1	29
1	1	18
1	1	21
3	1	23
1	1	23
1	1	26
3	3	23
1	3	25
2	2	22
2	1	31
1	1	27
1	1	20
3	1	21
1	1	20
1	1	27
1	1	23
1	1	17
3	1	31
1	1	23
1	1	33
1	1	21
2	1	22
1	1	23
2	1	19
2	2	19
3	2	23
1	1	23
1	1	31
1	1	21
1	1	30
1	1	18
1	1	21
1	1	27
4	4	23
2	1	31
1	1	23
1	1	27
1	1	18
1	1	19
1	2	23
1	1	30

ZB	CAUTI	UCR-MPI	Frequency of external c	Unplanned extubation rate
1	1	0	3	0
1	2	0	1	0
1	1	0	1	0
1	1	0	2	0
1	1	0	4	0
1	1	0	3	0
1	1	0	1	0
1	1	0	2	0
1	2	0	2	0
1	1	1	3	0
1	1	0	1	0
1	1	0	3	0
1	1	0	5	0
1	1	0	5	0
1	1	0	0	0
1	1	0	4	0
1	1	0	3	0
1	1	0	2	0
1	1	0	6	0
1	1	0	3	0
1	2	0	3	0
1	1	0	2	0
1	1	0	2	0
1	1	0	4	0
1	1	0	6	0
1	1	0	2	0
1	1	0	1	0
1	1	0	1	0
1	1	0	0	0
1	1	0	3	0
1	1	0	3	0
1	2	1	2	0
1	1	0	5	0
1	1	0	3	0
1	1	0	1	0
1	1	1	1	0
1	1	0	3	0
1	1	0	4	0
1	1	1	3	0
1	1	0	2	0
1	1	1	2	0
1	1	0	5	0
1	1	0	2	0
1	1	0	2	0
1	1	0	4	0
1	1	1	3	0
1	1	0	3	0
1	1	0	4	0
1	1	0	5	0
1	1	0	3	0
1	1	0	3	0
1	1	0	1	0
1	1	0	6	0
1	1	0	4	0
1	1	0	3	0

1	1	0	3	0
1	1	0	2	0
1	1	0	3	0
1	1	0	3	0
1	1	0	3	0
1	2	0	0	0
1	1	0	1	0
1	1	0	1	0
1	1	0	1	0
1	1	0	6	0
1	1	0	1	0
1	1	0	1	0
1	1	0	4	0
1	1	0	4	0
1	1	0	4	0
1	1	0	2	0
1	1	1	3	0
1	1	0	5	0
1	1	0	5	0
2	1	0	2	0
2	1	0	2	0
2	2	0	0	0
2	1	0	2	0
2	1	0	6	0
2	2	0	4	0
2	1	0	1	0
2	1	0	4	0
2	1	0	4	0
2	1	0	3	0
2	2	0	2	0
2	1	0	2	0
2	1	0	0	0
2	1	0	1	0
2	1	1	4	0
2	1	0	2	0
2	1	0	6	0
2	1	0	3	0
2	1	0	6	0
2	1	0	1	0
2	1	0	0	0
2	1	0	2	0
2	1	0	2	0
2	1	0	4	0
2	1	0	2	0
2	1	0	1	0
2	1	0	2	0
2	1	0	2	0
2	1	1	4	0
2	1	0	1	0
2	1	0	3	0
1	2	0	2	0
1	1	0	0	0
1	1	0	0	0
1	1	0	0	0
1	1	0	1	0
1	1	0	0	0

1	1	0	0	0
1	1	0	0	0
1	1	0	0	0
1	1	0	0	0
1	1	0	2	0
1	1	0	0	0
1	1	0	0	0
1	1	0	0	0
1	1	0	0	0
1	1	0	0	0
1	1	0	1	0
1	1	0	0	0
1	1	0	0	0
1	1	0	0	0
1	1	0	0	0
1	1	0	0	0
1	1	0	2	0
1	1	0	1	0
1	1	0	0	0
1	1	0	0	0
1	1	0	0	0
1	1	0	0	0
1	1	0	0	0
1	1	0	0	0
1	1	0	0	0
1	1	0	0	0
1	1	0	0	0
1	1	0	0	0
1	1	0	1	0
2	1	0	4	0
2	1	0	2	0
2	1	0	6	0
2	1	0	1	0
2	1	0	3	0
2	2	0	2	0
2	1	0	4	0
2	2	1	2	0
2	2	0	3	0
2	1	0	3	0
2	1	0	3	0
2	2	0	2	0
2	1	0	1	0
2	1	0	3	0
2	1	0	1	0
2	2	0	0	0
2	1	0	0	0
2	1	0	0	0
2	1	0	0	0
2	1	0	0	0
2	1	0	1	0
2	1	0	1	0
2	1	0	0	0
2	1	0	2	0
2	1	0	0	0
2	1	0	0	0
2	1	0	1	0

2	1	0	2	0
2	1	0	0	0
2	1	0	0	0
2	1	0	0	0
2	1	0	0	0
2	1	0	0	0
2	1	0	1	0
2	1	0	0	0
2	1	0	0	0
2	1	0	0	0
2	1	0	1	0
2	1	0	1	0
2	1	0	1	0
2	1	0	0	0
2	1	0	2	0
2	1	0	1	0
2	1	0	0	0
2	1	0	0	0
2	1	0	0	0
2	1	0	0	0
2	1	0	0	0
2	1	0	0	0
2	1	0	0	0
2	1	0	1	0
2	1	0	1	0
2	1	0	0	0
2	1	0	2	0
2	1	0	0	0
2	1	0	0	0
2	1	0	0	0
2	1	0	0	0
2	1	0	0	0
2	1	0	1	0
2	1	0	3	0
2	1	0	0	0
2	1	0	0	0
2	1	0	0	0
2	1	0	0	0
2	1	0	0	0
2	1	0	0	0
2	1	0	0	0
